# Accelerometry-Based Machine Learning Estimation of Energy Expenditure and Physical Activity Intensity Classification in Daily Tasks

**DOI:** 10.3390/s26216996

**Published:** 2026-11-05

**Authors:** Daniela Oliveira, Lucas Veras, Hélder Fonseca

**Affiliations:** 1 Research Centre in Physical Activity, Health and Leisure (CIAFEL), Faculty of Sport, University of Porto, 4200-450 Porto, Portugal; up201203953@up.pt (D.O.); lveras@uams.edu (L.V.); 2 Laboratory for Integrative and Translational Research in Population Health (ITR), 4050-600 Porto, Portugal

**Keywords:** accelerometry, energy expenditure, physical activity intensity, machine learning, wrist dominance, indirect calorimetry, daily living activities

## Abstract

**Highlights:**

**What are the main findings?**

- Random forest models outperformed K-nearest neighbors in overall energy expenditure estimation and physical activity intensity classification.
- Dominant and non-dominant wrist placements showed similar energy expenditure estimation errors but markedly different intensity classification performance.

**What are the implications of the main findings?**

- The non-dominant wrist may be a suitable single accelerometer placement when both energy expenditure and physical activity intensity are outcomes of interest.
- Dominant and non-dominant wrist placements should be clearly distinguished and consistently reported in accelerometry studies.

**Abstract:**

Accelerometers are widely used to estimate energy expenditure (EE) and classify physical activity intensity (PAI), although conventional approaches may not capture the complexity of raw acceleration signals. This study evaluated machine learning (ML) models for estimating EE and classifying PAI during daily and locomotor tasks. Forty-nine adults (28.4 ± 8.5 years) completed seven laboratory activities while wearing ActiGraph GT9X Link accelerometers on the right hip and dominant and non-dominant wrists. EE was measured by indirect calorimetry. Time- and frequency-domain accelerometry features from non-overlapping 1-s windows were combined with age, sex, body mass index (BMI), and body mass. K-nearest neighbors (KNN) and random forest (RF) models were developed for each placement using an 80/20 training–test split. RF achieved lower overall EE estimation errors and higher overall PAI classification agreement than KNN across placements. The lowest EE errors were obtained from RF models at the non-dominant wrist (RMSE = 0.67 kcal·min^−1^; MAE = 0.39 kcal·min^−1^) and dominant wrist (RMSE = 0.68 kcal·min^−1^; MAE = 0.40 kcal·min^−1^). RF classification agreement reached 95.81% (Kappa = 0.943) at the non-dominant wrist and 94.97% (Kappa = 0.931) at the hip. In this laboratory protocol, the non-dominant wrist RF model showed a favorable combination of EE estimation error and PAI classification agreement.

## 1. Introduction

In an era characterized by increasingly sedentary lifestyles and a rising burden of chronic non-communicable diseases, promoting physical activity is a crucial strategy for improving public health and well-being [1]. Physical activity encompasses a wide spectrum of bodily movements, from structured exercise to daily tasks such as walking, stair climbing, and household chores. Its health benefits are well documented [2,3], and are influenced by the volume and intensity of the movement performed [4]. Accurate assessment of physical activity intensity (PAI) and energy expenditure (EE) is essential for characterizing movement patterns and understanding their relationship with health outcomes [5]. However, quantifying these outcomes across a wide range of daily activities remains challenging, as human movement includes diverse and often complex motor patterns, which may differ substantially in their metabolic demands [6].

Several methods for PAI and EE assessment are available, each with its own limitations. Subjective methods, such as questionnaires, are cost-effective and feasible for large samples but are susceptible to recall and reporting bias, and may therefore provide imprecise estimates [7,8]. Among objective methods, doubly labeled water provides accurate estimates of total EE under free-living conditions but does not determine the energy cost of individual activities [9]. In contrast, indirect calorimetry allows EE to be measured during specific activities, although the equipment and procedures involved limit its practicality for prolonged monitoring in everyday settings [10]. Both methods require specialized equipment or analytical resources, which may limit their use in large-scale studies [11,12].

Recent technological advances have broadened the approaches to assessing PAI and EE [11,13,14]. Accelerometers are among the most commonly used wearable devices to assess physical activity patterns and intensity levels [5] and offer a unique opportunity to bridge the gap between laboratory-based assessments and the highly dynamic nature of daily activities [13]. Accelerometer output can be expressed as raw acceleration (typically in gravitational units, *g*) or as activity counts derived through manufacturer-specific processing procedures that are not always fully disclosed and that can limit comparisons between different brands and devices [14,15,16,17,18]. Although raw acceleration allows more transparent processing, calibration is still required to convert acceleration into biologically meaningful outcomes, such as EE [19,20,21].

Regression equations and intensity cut-points have traditionally been used to estimate EE and classify PAI from accelerometer data since they are relatively simple to implement and interpret [7]. However, these approaches often rely on a small set of summary metrics, which may not fully capture the temporal and frequency-domain characteristics of raw acceleration [22]. Their performance can also vary according to the activities included in the calibration protocol, accelerometer placement, and population characteristics [21,23].

Machine learning (ML) methods provide a compelling alternative for processing accelerometer data. These methods can integrate multiple features extracted from raw acceleration signals, including time- and frequency-domain features, and combine them with subject characteristics (e.g., age, sex, and anthropometric measures). ML models can also capture non-linear relationships and interactions between predictors that may not be adequately represented by conventional regression equations [24,25]. Random forests (RF), artificial neural networks, support vector machines, gradient boosting, and k-nearest neighbors (KNN) have previously been applied to estimate EE and classify PAI from wearable sensor data [24,26,27,28,29]. Recent studies have reported favorable performance of RF in EE estimation [28], while KNN has also demonstrated promising results for accelerometry-based PAI classification [30]. These findings provide a rationale for comparing RF, an established ensemble-learning method, with KNN, a relatively simple distance-based method, for both EE estimation and PAI classification.

Furthermore, accelerometer placement is an important methodological consideration, as devices worn at different anatomical sites capture distinct aspects of body movement that may influence model performance [24,31,32]. Previous studies have compared EE estimation across the hip, thigh, and both wrists [25], as well as PAI classification across these anatomical placements [33,34]. Differences in free-living accelerometry-derived outcomes have also been observed between the dominant and non-dominant wrists [35]. Although EE estimation and PAI classification have been investigated across different accelerometer placements, many of these studies used a single wrist, typically the non-dominant wrist, or addressed only one outcome, and the combined evaluation of both outcomes using hip, dominant-wrist, and non-dominant-wrist data from the same participants and experimental protocol remains limited. This comparison is of practical relevance because the wrist on which devices are worn varies across studies and consumer wearables, and because upper-limb movement during daily tasks may differ between the dominant and non-dominant arms. Such comparisons can also show whether accelerometer placement influences continuous EE estimation and categorical PAI classification in a similar manner.

Therefore, this study aimed to develop and evaluate RF and KNN models to estimate EE and classify PAI during daily living and locomotor activities, using features derived from raw accelerometer data collected simultaneously from the hip, dominant and non-dominant wrists, and to compare model performance across placements and activities.

## 2. Materials and Methods

### 2.1. Participants

Forty-nine adults (28 males; 28.4 ± 8.5 years, range 18–57 years; 168.7 ± 8.3 cm; 69.4 ± 13.5 kg; body mass index (BMI): 24.5 ± 3.7 kg·m^−2^; mean ± SD) were recruited from the local university campus through online advertisements. Height was measured using a stadiometer (Seca 213, Hamburg, Germany), and body mass with a digital scale (Seca 899, Hamburg, Germany) following standard procedures [36]. Inclusion criteria were age between 18 and 65 years; no self-reported neurological or musculoskeletal limitations that could compromise the performance of the protocol tasks; and familiarity with the protocol activities, specially walking and running on a treadmill. Exclusion criteria were conditions potentially affecting EE (e.g., recent body mass change > 10%, hypo- or hyperthyroidism), current use of any medication that could significantly affect EE (e.g., stimulants, beta-adrenergic or anticholinergic drugs, thyroid hormones) or any health condition that could contraindicate the performance of the assessments included in the protocol (e.g., pregnancy, phobias). A questionnaire administered at the time of the assessment collected information on lifestyle factors (smoking, alcohol consumption, PA/exercise, sleep duration, last meal, fasting time) as well as current medication. Participants were instructed to fast overnight for at least 12 h (while remaining hydrated with water), to avoid alcoholic beverages and coffee in the preceding 12 h, to refrain from vigorous PA in the preceding 24 h, to refrain from smoking on the day of the assessment, to obtain adequate sleep, and to empty their bladder before the assessment. All participants were informed about the purpose and protocol of the study before giving written informed consent. The study protocol was approved by the local Ethics Committee (CES 192-14).

### 2.2. Protocol and Measurements

The protocol was divided in two parts. In the first part, participants lay supine at rest for 30 min to assess resting EE through indirect calorimetry (Quark CPET, Cosmed, Rome, Italy). During this measurement, participants remained alone in a quiet, darkened room at an ambient temperature of 22 °C. Resting EE was obtained from the 10 consecutive minutes with the lowest variation in EE and respiratory exchange ratio (RER), selected as follows: after the first 5 min were discarded to allow familiarization, a 10-min moving window (advanced in 1-min steps) was applied to the remaining data, and the window with the lowest mean of the coefficients of variation (CV) of EE and RER was selected. Steady state was confirmed when the CV was ≤10% for EE and ≤5% for RER. Oxygen uptake ($\dot{V}O_{2}$) and carbon dioxide production ($\dot{V}{\mathrm{CO}}_{2}$) were used to calculate EE according to Weir’s equation: $\mathrm{EE}\left(\mathrm{kcal}\cdot \min^{-1}\right)=3.941\times \dot{V}O_{2}+1.106\times \dot{V}{\mathrm{CO}}_{2}$, where $\dot{V}O_{2}$ and $\dot{V}{\mathrm{CO}}_{2}$ are expressed in L∙min^−1^ [37].

In the second part of the protocol, participants performed seven pre-established activities, for five minutes each, with one-minute rest intervals between activities. The tasks were as follows: (1) sitting and typing on a computer; (2) standing and transferring water from one basin to another; (3) household chores: carrying bags with fractional weights (2, 1.5, 1, and 0.5 kg) and organizing them, dusting shelves, sweeping the floor with a broom, picking up the trash, storing and arranging a set of sheets and books; (4) slow walking at 4 km·h^−1^; (5) fast walking at 6 km·h^−1^; (6) running at 9 km·h^−1^; and (7) climbing stairs. During the household chores, participants performed the subtasks in the specified order at a self-selected pace, repeating the sequence if completed before the end of the five-minute period. No instructions were given regarding hand use, so that participants performed the tasks as they would in daily life. During the stair task, participants repeatedly walked up and down two flights of stairs (step height, 16 cm) at a self-selected pace for five minutes. All walking and running tasks were performed on a treadmill (quasar 4.0; h/p/cosmos, Nussdorf-Traunstein, Germany). During these activities, EE was continuously measured using a portable indirect calorimetry system (Cosmed K5, Cosmed, Rome, Italy) worn on the participant’s back and connected to a face mask covering the nose and mouth, which recorded VO_2_ and VCO_2_ breath-by-breath. Before each session, the K5 was calibrated according to the manufacturer’s instructions, including room-air and reference-gas calibration, and flowmeter calibration with a 3-L syringe.

While performing the activities, participants also wore three accelerometers (GT9X Link, 100 Hz; ±16 *g* range; ActiGraph, Pensacola, FL, USA) placed as follows: (i) right hip (along the anterior axillary line, at the level of the iliac crest); (ii) non-dominant wrist, and (iii) dominant wrist, both at the level of the styloid process of the ulna. Hand dominance was determined by self-report of the hand used for writing. Of all participants, 46 reported being right-handed and 3 left-handed. A member of the research team verified accelerometer placement before each task. Accelerometer and respiratory gas data were collected throughout all activities. All assessments were performed in the morning between 9:00 and 11:00 a.m.

### 2.3. Data Processing and Statistical Analysis

The criterion EE and PAI label assigned to each 1-s window were derived as follows: EE was calculated for each breath using Weir’s equation [37], then the breath-by-breath EE was linearly interpolated to 1-s intervals and aligned with the accelerometer windows using the timestamps of both devices. After that, each 1-s window was assigned an intensity class based on metabolic equivalents (METs), that were calculated individually as the ratio of the EE of each window to the participant’s resting EE. The thresholds used to establish PAI were: sedentary, <1.5 METs; light, ≥1.5 and <3.0 METs; moderate, ≥3.0 and <6 METs; and vigorous, ≥6.0 METs. To reduce the influence of transient responses, the first and last minute of each protocol task were excluded, except for task 3, which was a circuit involving multiple activities. Thus, most observations were drawn from the central portion of each task, which was intended to represent steady-state performance.

For PAI classification and EE prediction, two supervised ML algorithms were implemented: KNN and RF. These algorithms were selected because they represent two contrasting non-parametric approaches (instance-based and a tree-based ensemble methods, respectively), that have been widely used for accelerometer-based EE estimation and PAI classification [27,28,29]. Both can model non-linear relationships without assuming specific data distributions, and are relatively straightforward to implement and tune. For EE estimation, the outcome was the numerical value of physical activity EE expressed in kcal·min^−1^, with resting energy expenditure being subtracted from the total energy expenditure. For PAI classification, the outcome variable was a categorical variable with four classes (sedentary, light, moderate, and vigorous).

Models were developed separately for each accelerometer placement (hip, non-dominant wrist, and dominant wrist). Each placement contributed 54 features derived from the triaxial (x, y, z) raw acceleration signal, encompassing both time- and frequency-domain measures. Features were extracted using non-overlapping 1-s windows. For each accelerometer axis, 12 time-domain features were calculated: mean, standard deviation, coefficient of variation, minimum, maximum, 25th percentile, median, 75th percentile, skewness, kurtosis, mean absolute amplitude, and standard deviation of absolute amplitude. Four frequency-domain features were calculated for each axis: dominant frequency, median frequency, dominant magnitude, and total power. Three inter-axis correlations (xy, xz, yz) and three orientation-related variables were also computed, yielding 54 accelerometer-derived features per placement. These features were combined with four demographic variables (age, sex, BMI, and body mass), yielding 58 variables before preprocessing. A dictionary describing all accelerometer-derived features is provided in Supplementary File S1.

Because multiple 1-s observations were obtained from each participant, the participant was treated as the independent unit for model validation. Therefore, participants, rather than individual observation windows, were split into training (80%) and testing (20%) sets [34], with stratified sampling applied for classification to maintain class proportions. All observations from a given participant were assigned to the same partition. In total, 10 participants were included in the testing set, with a total of 20,258 windows. The same test participants were used for all accelerometer placements and ML models.

Predictor preprocessing included median imputation, normalization by centering and scaling, and removal of near-zero-variance predictors. Within each cross-validation fold, these preprocessing parameters were estimated using only the training participants and then applied to the validation participants. Hyperparameters were tuned using grouped 5-fold cross-validation, with folds defined by participant and the same fold assignments used across placements and models. For the final models, preprocessing was refit using the full training set and then applied to the held-out participants. In these final fits, near-zero-variance filtering removed the *y*-axis dominant- and median-frequency features from the hip and nondominant-wrist models; no predictors were removed from the dominant-wrist models. For RF, the number of randomly selected predictors at each split (mtry) and the minimum node size (min_n) were tuned, with the number of trees fixed at 500. For KNN, the number of neighbors (k) was tuned. In both cases, a grid of 10 hyperparameter combinations was evaluated, and the best combination was selected based on accuracy for classification and root mean squared error (RMSE) for regression. Final models were then fit on the entire training set using the selected hyperparameters. The trained final models are provided in Supplementary File S2.

Model performance was assessed overall, and also by activity. For the EE estimation models, performance was assessed using RMSE and mean absolute error (MAE), and also through Bland–Altman plots. Furthermore, proportional bias was assessed separately for each model and accelerometer placement using a linear mixed-effect model with difference between actual and predicted EE as the outcome, mean of actual and predicted EE as a fixed-effect predictor, and a random intercept for participant. The fixed-effect slope and its associated *p*-value were used to assess whether error varied with EE magnitude. As for the PAI classification, performance was evaluated using sensitivity, specificity, percent agreement, and Cohen’s Kappa coefficient. Within each activity, sensitivity and specificity were calculated separately for each intensity class using a one-versus-rest approach. Activity-specific values are unweighted averages across the four intensity classes. All analyses were conducted in R (version 4.5.1; R Foundation for Statistical Computing, Vienna, Austria) using the tidymodels framework (version 1.3.0; Posit PBC, Boston, MA, USA).

## 3. Results

### 3.1. EE Estimation

The performance of the KNN and RF models for EE estimation according to accelerometer placement is presented in Table 1. Across all placements, RF models yielded lower overall RMSE and MAE values than their corresponding KNN models. For the KNN models, performance was relatively similar across accelerometer placements, with RMSE values ranging from 1.02 to 1.10 kcal·min^−1^ and MAE values ranging from 0.64 to 0.68 kcal·min^−1^. The lowest RMSE was observed for the non-dominant wrist, while the MAE was the same for the dominant and non-dominant wrist placements.

For the hip placement, the RF model had an RMSE of 1.00 kcal·min^−1^ and an MAE of 0.64 kcal·min^−1^. Lower error values were obtained for both wrist placements. The dominant wrist model yielded an RMSE of 0.68 kcal·min^−1^ and an MAE of 0.40 kcal·min^−1^, while the non-dominant wrist model showed an RMSE of 0.67 kcal·min^−1^ and an MAE of 0.39 kcal·min^−1^. The RF model using data from the non-dominant wrist had numerically the lowest estimation errors.

**Figure 1:**
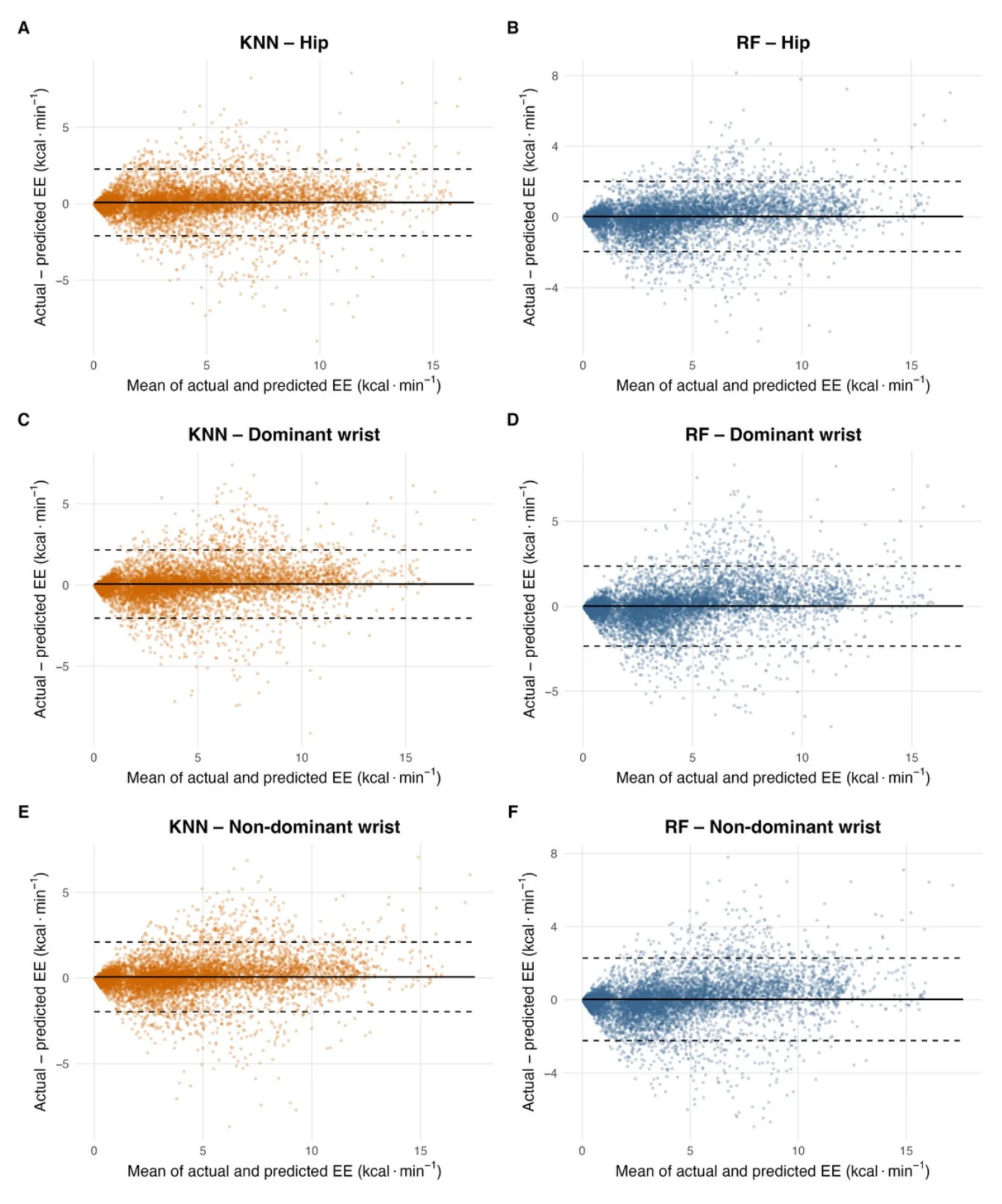
Bland–Altman plots comparing measured and predicted EE for the KNN and RF models according to accelerometer placement: (**A**) hip, KNN; (**B**) hip, RF; (**C**) dominant wrist, KNN; (**D**) dominant wrist, RF; (**E**) non-dominant wrist, KNN; and (**F**) non-dominant wrist, RF. The solid horizontal line represents the mean bias, and the dashed horizontal lines represent the 95% limits of agreement.

Bland–Altman plots for all models and accelerometer placements are shown in Figure 1. The dispersion of the differences increased as mean EE increased, indicating greater variability in estimation error at higher EE values. This pattern was observed for both algorithms and across the three accelerometer placements. Proportional bias was positive for all models and placements, with estimation error increasing, even though slightly, as the magnitude of EE increased (all *p* < 0.001). The Bland–Altman summary statistics are presented in Table 2.

### 3.2. Physical Activity Intensity Classification

The performance of the models for the classification of physical activity intensity is presented in Table 3. Overall, RF provided higher percent agreement and Kappa coefficients than KNN across the three accelerometer placements. However, substantial differences in performance were observed between placements.

For the hip placement, the KNN model achieved an overall percent agreement of 76.63% and a Kappa coefficient of 0.683. Sensitivity ranged from 0.622 for light-intensity activity to 0.883 for vigorous-intensity activity, while specificity ranged from 0.889 to 0.967. The corresponding RF model demonstrated better performance, achieving an overall percent agreement of 80.41% and a Kappa coefficient of 0.733. Sensitivity ranged from 0.694 to 0.885, and specificity ranged from 0.891 to 0.970 across the four intensity categories.

Classification performance was lower for models based on dominant-wrist acceleration. The KNN model achieved an overall percent agreement of 53.87% and a Kappa coefficient of 0.369, with sensitivity values ranging from 0.512 to 0.561 and specificity values ranging from 0.672 to 0.965. The RF model provided a modest improvement in overall performance, with a percent agreement of 59.24% and a Kappa coefficient of 0.438. Nevertheless, sensitivity remained relatively low across the intensity categories, ranging from 0.454 for sedentary activity to 0.667 for light-intensity activity.

Models based on the non-dominant wrist showed higher classification performance than those based on the dominant wrist. The non-dominant wrist KNN model achieved an overall percent agreement of 83.78% and a Kappa coefficient of 0.779. Sensitivity ranged from 0.740 to 0.902, whereas specificity ranged from 0.924 to 0.975. The highest overall classification performance was obtained by the RF model developed using non-dominant wrist data, which achieved a percent agreement of 95.81% and a Kappa coefficient of 0.943. Sensitivity ranged from 0.933 for light-intensity activity to 0.973 for vigorous-intensity activity, while specificity ranged from 0.977 to 0.995. Overall, RF models based on hip and non-dominant wrist acceleration provided similarly high classification performance, whereas both models developed using dominant-wrist data demonstrated substantially lower agreement with the criterion intensity classifications.

**Figure 2:**
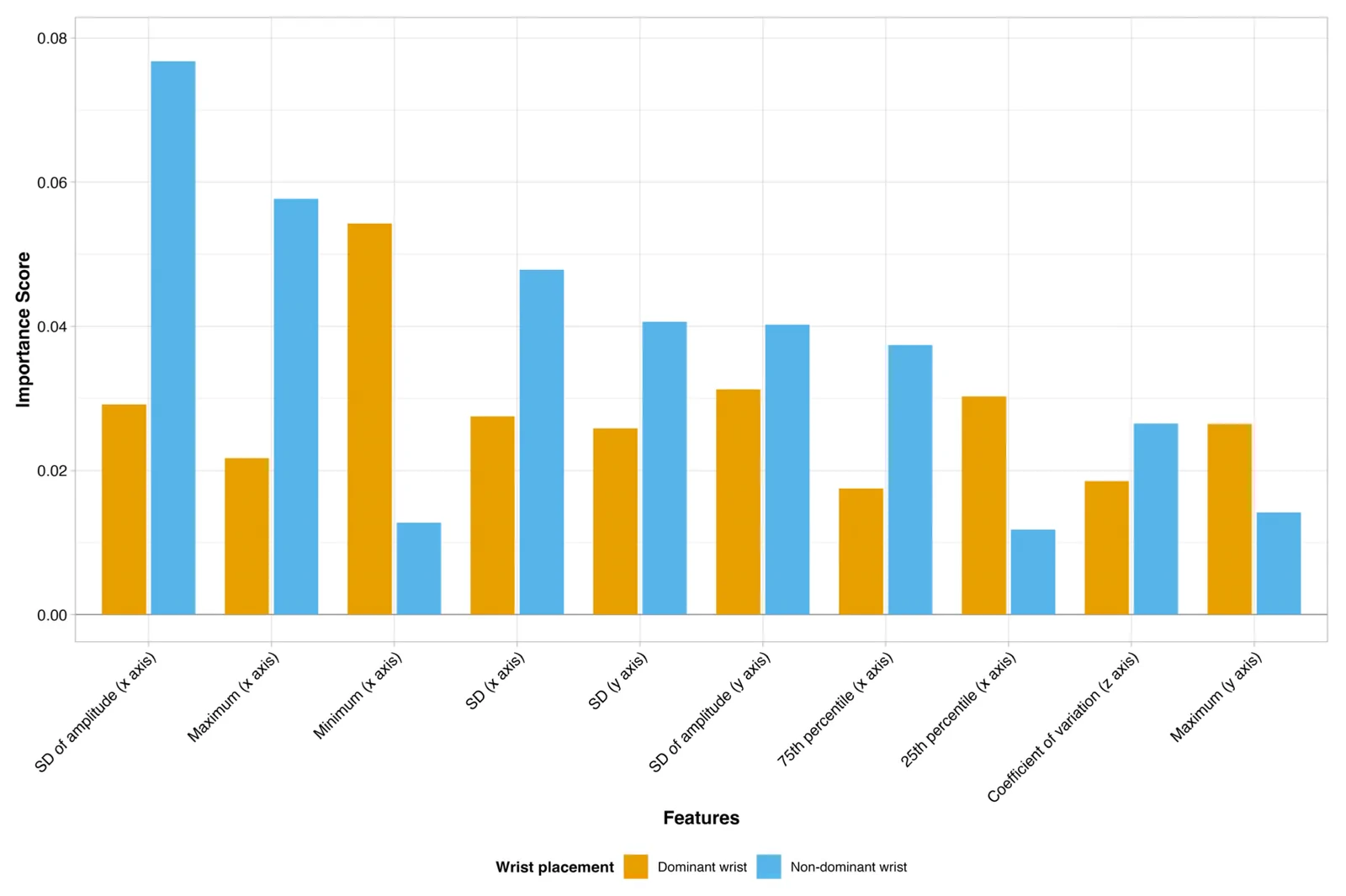
Random forest permutation-importance scores for physical activity intensity classification using dominant- and non-dominant-wrist accelerometer data. The figure shows the 10 acceleration features with the highest scores across either wrist. The importance scores indicate the relative contribution of each feature to the classification of physical activity intensity, with higher scores representing greater importance.

Due to the substantial differences found in PAI classification performance between the dominant and non-dominant wrists, we examined RF permutation importance to identify which acceleration features contributed to each wrist’s intensity classifications. Figure 2 shows the feature importance analysis using the RF algorithm. The dominant-wrist model ranked minimum *x*-axis acceleration highest, while the non-dominant-wrist model ranked *x*-axis signal-amplitude variability highest. Variability and amplitude features appeared prominently in both sets of top-ranked features. The rankings were strongly correlated across the 52 acceleration features shared by both wrists (Spearman ρ = 0.85), although five of the top ten features overlapped. This suggests a broadly similar pattern of influential features alongside some wrist-specific differences.

### 3.3. Activity-Specific EE Estimation

Activity-specific EE estimation errors are presented in Table 4. Across placements and models, the lowest estimation errors were generally observed during sitting and standing, whereas running and stair climbing showed the highest RMSE and MAE values.

For the hip placement, RMSE values ranged from 0.40 to 1.67 kcal·min^−1^ for KNN and from 0.36 to 1.52 kcal·min^−1^ for RF. The corresponding MAE values ranged from 0.26 to 1.11 kcal·min^−1^ for KNN and from 0.23 to 1.12 kcal·min^−1^ for RF. The largest errors for both hip-based models were observed during running and stair climbing. At the hip, RF did not outperform KNN for every activity: KNN yielded slightly lower errors during standing (RMSE, 0.47 vs. 0.49 kcal·min^−1^) and slow walking (RMSE, 0.63 vs. 0.66 kcal·min^−1^; MAE, 0.44 vs. 0.47 kcal·min^−1^), and a similar MAE during stair climbing (1.11 vs. 1.12 kcal·min^−1^). At both wrists, RF yielded lower errors than KNN for all activities.

Models based on dominant-wrist acceleration generally provided lower activity-specific estimation errors than hip-based models. The dominant-wrist KNN model presented RMSE values ranging from 0.34 to 1.67 kcal·min^−1^ and MAE values ranging from 0.24 to 1.20 kcal·min^−1^. For the dominant-wrist RF model, RMSE ranged from 0.22 kcal·min^−1^ during sitting and typing to 1.10 kcal·min^−1^ during stair climbing, while MAE ranged from 0.14 to 0.74 kcal·min^−1^.

Similar results were observed for the non-dominant wrist. The KNN model presented RMSE values ranging from 0.35 to 1.62 kcal·min^−1^ and MAE values ranging from 0.23 to 1.20 kcal·min^−1^. The non-dominant wrist RF model had RMSE values between 0.25 and 1.01 kcal·min^−1^ and MAE values between 0.14 and 0.69 kcal·min^−1^.

Among the RF models, the dominant wrist yielded the lowest RMSE for sitting, standing, fast walking, and running, whereas the non-dominant wrist had the lowest or similar errors for household chores, slow walking, and stair climbing. Activity-specific results were therefore consistent with the overall performance metrics, indicating lower estimation errors for RF models based on wrist accelerometer data.

### 3.4. Activity-Specific Physical Activity Intensity Classification

The activity-specific performance of the PAI classification models is presented in Table 5. For the hip placement, percent agreement for the KNN model ranged from 55.6% during household chores to 90.5% during fast walking, while Kappa coefficients ranged from 0.223 to 0.549. For the RF model, percent agreement ranged from 69.1% during household chores to 90.2% during fast walking, with Kappa coefficients ranging from 0.405 to 0.581.

For the dominant wrist, percent agreement for the KNN model ranged from 38.8% during stair climbing to 72.4% during fast walking, while Kappa coefficients ranged from −0.034 to 0.176. For the RF model, percent agreement ranged from 44.1% during stair climbing to 78.3% during fast walking, with Kappa coefficients ranging from 0.068 to 0.227.

Higher activity-specific classification performance was observed for the non-dominant wrist. For the KNN model, percent agreement ranged from 73.9% during household chores to 92.8% during fast walking, while Kappa coefficients ranged from 0.520 to 0.679. The RF model using non-dominant wrist data achieved the highest activity-specific classification performance across all tasks. Percent agreement ranged from 93.3% during household chores to 98.0% during fast walking. Kappa coefficients ranged from 0.873 for to 0.920.

## 4. Discussion

This study identified differences in the performance of KNN and RF models for EE estimation and PAI classification across accelerometer placements. RF models yielded lower overall EE estimation errors than KNN models across all accelerometer placements. The lowest RMSE and MAE values were obtained from the wrist-based RF models, with similar results for the dominant and non-dominant wrists. For PAI classification, the hip and non-dominant wrist RF models showed the highest overall agreement. However, the activity-specific results favored the non-dominant wrist across the seven tasks.

The RF models showed slightly lower EE estimation errors compared to the KNN models. However, this was not observed for every activity, as KNN models showed similar or slightly lower errors in some conditions, such as the hip-worn accelerometers on standing and slow walking activities, where movement patterns are relatively simple and between-model differences were small. The difference between RMSE and MAE indicates that, although most errors were small, larger errors occurred in a subset of observations with higher EE, consistent with what was shown in the Bland–Altman plots, and the tendency to overestimate EE in the upper measurement range.

One possible explanation for the better RF performance is its capacity to model non-linear relationships and interactions among the time- and frequency-domain acceleration features and participant characteristics, whereas nearest-neighbor distances may be less informative when activities have overlapping acceleration profiles. This interpretation is consistent with Montoye et al. [25], who found that non-linear models showed greater improvements for wrist-worn than hip- or thigh-worn accelerometers. Similarly, the reduction in RMSE from KNN to RF was small at the hip in the present study but more pronounced at both wrists, suggesting that wrist-derived features may benefit from models that capture complex relationships among signal characteristics.

Previous studies support the value of ML approaches for estimating EE and classifying PAI from accelerometer data. O’Driscoll et al. [27] reported that ML models could improve EE estimation across different activity types, which provides context for the lower overall EE estimation errors observed for RF in the present study. Similar advantages of ML over linear regression equations and cut-points have been reported for EE estimation and PAI classification from hip and wrist accelerometers [31,38].

Activity-specific EE estimation errors were lower during sitting, standing, and walking and higher during running, stair climbing, and household chores. Running and stair climbing involve inter-individual differences in movement economy, stride pattern, vertical displacement, and metabolic response that are not fully represented by acceleration from one anatomical location. Household chores posed a different challenge: their non-cyclical, asymmetric, and self-paced movements can be performed using different strategies, which may affect metabolic cost. These findings highlight the importance of evaluating models across a range of movements beyond structured locomotion.

For PAI classification, the dominant-wrist models had lower sensitivity, agreement, and Kappa than the other placements. Their relatively high specificity should be interpreted cautiously because, in a one-versus-rest multiclass evaluation, specificity primarily reflects the correct rejection of observations belonging to the remaining classes and can therefore remain high even when a category is not identified well [39].

One result that should be noted is the difference in PAI classification performance between dominant and non-dominant wrists. Nonetheless, this finding is consistent with prior evidence that placing accelerometers in different wrists can affect intensity classification. Montoye et al. [34] found higher sensitivity and specificity for PAI classification from left wrist accelerometers (which were worn on the non-dominant wrist by 90% of their participants) than from the right wrist, and these differences were particularly evident for MVPA. The authors suggested that greater movement variability of the dominant hand might contribute to this difference. In our analysis, the models showed a broadly similar pattern of feature importance across wrists for the RF models, although the relative contribution of individual features differed, suggesting that they relied on overlapping but not identical acceleration information. A potential explanation is that, during sedentary activities involving manual tasks such as object manipulation, movement of the dominant hand may exceed that of the non-dominant hand, producing wrist-acceleration differences that reflect task-related arm movement rather than whole-body activity intensity.

Furthermore, the high overall agreement of the hip RF model for PAI classification may reflect that hip acceleration is less influenced by isolated upper-limb movements and represents trunk displacement more directly during walking and running. Taken together, the results do not identify a universally superior placement. Within the population and protocol studied, the non-dominant wrist provided the most consistent performance across both outcomes, whereas in other contexts the most appropriate placement may depend on whether the primary objective is continuous EE estimation, intensity classification, or both.

This study has limitations that should be acknowledged. First, the controlled laboratory protocol included only a limited range of daily-life movement patterns, whereas free-living activities are often shorter, interrupted, combined with other tasks, and self-paced. Consequently, performance may be lower for activities that were not represented for model training, which limits the external validity of the study. Second, the sample was relatively small, with 49 predominantly young, healthy adults, with BMI near the normal range. The findings may not generalize to children, older adults, people with obesity, or clinical populations with altered gait, movement economy, or metabolic responses. Validation in larger and more heterogeneous cohorts is therefore required to broaden the study findings to other populations. Third, the 1-s non-overlapping windows captured rapid changes in acceleration, whereas the metabolic response measured by indirect calorimetry may lag behind changes in movement, particularly during transitions. Future work should evaluate alternative window durations and approaches that incorporate temporal context. Finally, as only two ML models were evaluated and no direct comparison with more traditional methods was made, our findings cannot definitively establish whether these models offer superior performance over conventional approaches to EE estimation and PAI classification. Therefore, more comprehensive studies testing other types of statistical models and ML algorithms are still needed. This study also has several strengths. EE was continuously measured through indirect calorimetry, providing an objective criterion during the activity protocol. Accelerometers were worn simultaneously on the hip, dominant wrist, and non-dominant wrist, allowing the three placements to be compared within the same participants and under the same experimental conditions. The direct comparison between both wrists was particularly relevant, as it showed that the effect of wrist side depended on the outcome.

The protocol combined structured walking and running with stair climbing and daily tasks involving variable and asymmetric movements. Including these tasks increased the complexity of the prediction problem and allowed performance to be evaluated across a wider range of movement patterns than a protocol restricted to treadmill locomotion alone. The models used several time- and frequency-domain features from the raw triaxial acceleration signal and were evaluated for two related but distinct outcomes. Reporting both overall and activity-specific performance made it possible to identify differences that would have been concealed by a single overall metric.

From a real-world application perspective, the robustness of the proposed models has not yet been established. Several factors not addressed in the present protocol may affect performance outside the laboratory, including variations in device orientation, and position at the wrist or hip; and the frequent transitions, short bouts, and combined or unstructured activities typical of daily life. On the other hand, the models rely on features derived from raw triaxial acceleration and on readily available participant characteristics, and trained RF models are computationally inexpensive to apply, which favors their implementation in large-scale or long-term monitoring. However, these results should still be tested through free-living validation against appropriate criterion measures, and in more heterogeneous populations.

## 5. Conclusions

This study developed and evaluated RF and KNN models for EE estimation and PAI classification using accelerometer data from the hip, dominant wrist, and non-dominant wrist during controlled daily-life and locomotor activities. RF models provided lower overall EE estimation errors and higher overall PAI classification agreement than KNN models. The wrist-based RF models yielded the lowest EE estimation errors, while the non-dominant wrist RF model showed the highest PAI classification agreement within the study protocol. These findings suggest that the influence of accelerometer placement may differ depending on the outcome being estimated and highlight the importance of standardizing wrist placement in relation to hand dominance, given that the wrist on which devices are worn varies across studies and consumer wearables. However, the relatively small and predominantly young, healthy sample and the limited range of laboratory-based activities limit the generalizability of these findings. Further validation in larger and more heterogeneous populations and under free-living conditions is needed to determine whether comparable performance can be achieved outside laboratory conditions.

## Figures and Tables

**Table 1:** Performance of KNN and RF models for EE estimation by accelerometer placement.

Placement	Model	RMSE (kcal·min^−1^)	MAE (kcal·min^−1^)
Hip	KNN	1.10	0.68
	RF	1.00	0.64
Dominant Wrist	KNN	1.04	0.64
	RF	0.68	0.40
Non-dominant Wrist	KNN	1.02	0.64
	RF	0.67	0.39

Abbreviations: KNN, K-nearest neighbors; RF, random forest; RMSE, root mean square error; MAE, mean absolute error.

**Table 2:** Bland–Altman plots summary statistics (including bias, lower and upper limits of agreement, proportional bias and its associated *p*-value) of KNN and RF models for EE estimation by accelerometer placement.

Placement	Model	Bias (kcal·min^−1^)	Lower LoA (kcal·min^−1^)	Upper LoA (kcal·min^−1^)	Proportional Bias	*p*-Value
Hip	KNN	0.082	−2.101	2.266	0.0352	<0.001
	RF	0.026	−1.957	2.01	0.0325	<0.001
Dominant Wrist	KNN	0.066	−2.037	2.17	0.0399	<0.001
	RF	0.016	−2.343	2.374	0.0383	<0.001
Non-dominant Wrist	KNN	0.067	−1.970	2.103	0.0405	<0.001
	RF	0.022	−2.232	2.275	0.0354	<0.001

Abbreviations: KNN, K-nearest neighbors; RF, random forest; LoA, limits of agreement.

**Table 3:** Performance of KNN and RF models for PAI classification by accelerometer placement.

Placement	Model	Intensity Category	Sensitivity	Specificity	Percent Agreement	Kappa
Hip	KNN	Sedentary	0.854	0.926	76.63%	0.683
		Light	0.622	0.889		
		Moderate	0.760	0.899		
		Vigorous	0.883	0.967		
	RF	Sedentary	0.855	0.960	80.41%	0.733
		Light	0.694	0.908		
		Moderate	0.813	0.891		
		Vigorous	0.885	0.970		
Dominant Wrist	KNN	Sedentary	0.536	0.933	53.87%	0.369
		Light	0.561	0.672		
		Moderate	0.512	0.790		
		Vigorous	0.561	0.965		
	RF	Sedentary	0.454	0.959	59.24%	0.438
		Light	0.667	0.710		
		Moderate	0.636	0.805		
		Vigorous	0.557	0.959		
Non-dominant Wrist	KNN	Sedentary	0.902	0.950	83.78%	0.779
		Light	0.740	0.930		
		Moderate	0.844	0.924		
		Vigorous	0.892	0.975		
	RF	Sedentary	0.967	0.990	95.81%	0.943
		Light	0.933	0.980		
		Moderate	0.964	0.977		
		Vigorous	0.973	0.995		

Abbreviations: KNN, K-nearest neighbors; RF, random forest.

**Table 4:** Activity-specific performance of KNN and RF models for EE estimation by accelerometer placement.

Placement	Model	Metric (kcal·min^−1^)	Sitting Typing	Standing	Household Chores	Slow Walking	Fast Walking	Running	Stairs Climbing
Hip	KNN	RMSE	0.40	0.47	1.36	0.63	0.82	1.67	1.61
		MAE	0.26	0.33	1.00	0.44	0.59	1.10	1.11
	RF	RMSE	0.36	0.49	1.09	0.66	0.79	1.51	1.52
		MAE	0.23	0.31	0.80	0.47	0.57	1.03	1.12
Dominant Wrist	KNN	RMSE	0.34	0.47	1.21	0.64	0.75	1.56	1.67
		MAE	0.24	0.31	0.88	0.42	0.52	1.02	1.20
	RF	RMSE	0.22	0.34	0.78	0.45	0.53	1.00	1.10
		MAE	0.14	0.21	0.52	0.29	0.33	0.62	0.74
Non-dominant Wrist	KNN	RMSE	0.35	0.51	1.10	0.62	0.77	1.50	1.62
		MAE	0.23	0.33	0.81	0.42	0.53	0.99	1.20
	RF	RMSE	0.25	0.37	0.69	0.44	0.54	1.01	1.00
		MAE	0.14	0.21	0.46	0.29	0.34	0.61	0.69

Abbreviations: KNN, K-nearest neighbors; RF, random forest; RMSE, root mean square error; MAE, mean absolute error.

**Table 5:** Activity-specific performance of KNN and RF models for PAI classification by accelerometer placement.

Placement	Model		Sitting Typing	Standing	Household Chores	Slow Walking	Fast Walking	Running	Stairs Climbing
Hip	KNN	Sensitivity	0.471	0.488	0.317	0.451	0.488	0.426	0.558
		Specificity	0.853	0.867	0.812	0.869	0.861	0.847	0.895
		Percent agreement	79.5%	75.4%	55.6%	73.5%	90.5%	86.1%	76.0%
		Kappa	0.449	0.471	0.223	0.474	0.540	0.448	0.549
	RF	Sensitivity	0.536	0.531	0.370	0.446	0.465	0.389	0.540
		Specificity	0.878	0.899	0.852	0.882	0.845	0.838	0.898
		Percent agreement	83.1%	79.9%	69.1%	76.3%	90.2%	86.1%	78.1%
		Kappa	0.552	0.581	0.405	0.529	0.494	0.414	0.577
Dominant Wrist	KNN	Sensitivity	0.368	0.400	0.309	0.237	0.396	0.374	0.378
		Specificity	0.778	0.785	0.753	0.741	0.764	0.802	0.770
		Percent agreement	59.6%	50.9%	45.7%	47.2%	61.4%	72.4%	38.8%
		Kappa	0.096	0.116	0.011	−0.034	0.051	0.176	0.063
	RF	Sensitivity	0.360	0.435	0.273	0.316	0.393	0.529	0.406
		Specificity	0.772	0.787	0.770	0.793	0.790	0.816	0.770
		Percent agreement	57.3%	49.8%	53.1%	58.0%	78.3%	72.9%	44.1%
		Kappa	0.074	0.120	0.080	0.170	0.157	0.227	0.068
Non-dominant Wrist	KNN	Sensitivity	0.594	0.703	0.444	0.504	0.634	0.732	0.689
		Specificity	0.874	0.906	0.886	0.921	0.891	0.866	0.915
		Percent agreement	84.6%	82.0%	73.9%	83.8%	92.8%	88.6%	80.2%
		Kappa	0.559	0.619	0.520	0.679	0.656	0.547	0.628
	RF	Sensitivity	0.961	0.800	0.873	0.816	0.902	0.932	0.883
		Specificity	0.971	0.976	0.969	0.977	0.967	0.967	0.981
		Percent agreement	96.2%	95.0%	93.3%	95.3%	98.0%	97.1%	95.9%
		Kappa	0.904	0.895	0.873	0.907	0.912	0.904	0.920

Abbreviations: KNN, K-nearest neighbors; RF, random forest.

## Data Availability

The datasets presented in this article are not readily available because the data are part of an ongoing study. Requests to access the datasets should be directed to Lucas Veras.

## Supplementary Materials

The following supporting information can be downloaded at: https://www.mdpi.com/article/10.3390/s26216996/s1, File S1: Accelerometer feature dictionary; File S2: Trained Models. https://doi.org/10.5281/zenodo.21998043 (accessed on 30 October 2026).

## Abbreviations

The following abbreviations are used in this manuscript:

BMI	body mass index
EE	energy expenditure
KNN	K-nearest neighbors
MAE	mean absolute error
ML	machine learning
PA	physical activity
PAI	physical activity intensity
RF	random forest
RMSE	root mean squared error
