# Supplementary material for: Accelerometry-Based Machine Learning Estimation of Energy Expenditure and Physical Activity Intensity Classification in Daily Tasks
Source: Sensors (Basel). 2026 Nov 5;26(21):6996. doi: 10.3390/s26216996 (PMC13766557; doi:10.3390/s26216996)
Supplement: Supplementary file 1 [file sensors-26-06996-s001.zip › sensors-4544718-S1_Accelerometer_feature_dictionary.pdf]

Accelerometer feature dictionary

This dictionary defines the accelerometer-derived features calculated in non-overlapping 1-s windows for each placement (hip, non-dominant wrist, and dominant wrist). The 54 features comprise 36 time-domain features, 12 frequency-domain features, 3 correlations, and 3 orientation-related features. Age, sex, BMI, and body mass are four additional predictors, yielding 58 predictors before preprocessing.

Time-domain features

For each axis $q \in \{x, y, z\}$, let $a_{q,i}$ denote sample i in a window of N samples. The features below are calculated separately for each axis, yielding 36 time-domain features per placement. Feature names use the axis prefix, for example, `x_mean`, `y_mean`, or `z_mean`.

Table S1. Equations for time-domain features

Feature name pattern	Equation
`{q}_mean`	$\bar{a}_q = \frac{1}{N}\sum_{i=1}^{N} a_{q,i}$
`{q}_sd`	$s_q = \sqrt{\frac{1}{N-1}\sum_{i=1}^{N}(a_{q,i} - \bar{a}_q)^2}$
`{q}_cv`	$s_q/\bar{a}_q$
`{q}_min`	$\min_i a_{q,i}$
`{q}_max`	$\max_i a_{q,i}$
`{q}_q25`	25th percentile of $a_{q,1}, \dots, a_{q,N}$
`{q}_median`	50th percentile of $a_{q,1}, \dots, a_{q,N}$
`{q}_q75`	75th percentile of $a_{q,1}, \dots, a_{q,N}$
`{q}_skewness`	Sample skewness of $a_{q,1}, \dots, a_{q,N}$
`{q}_kurtosis`	Sample kurtosis of $a_{q,1}, \dots, a_{q,N}$
`{q}_mean_amplitude`	$\frac{1}{N}\sum_{i=1}^{N} \lvert a_{q,i}\rvert$
`{q}_sd_amplitude`	Sample SD of $\lvert a_{q,1}\rvert, \dots, \lvert a_{q,N}\rvert$

Frequency-domain features

The following four features are calculated separately for each axis $q \in \{x, y, z\}$, yielding 12 frequency-domain features per placement. Each axis is mean-centered and Hann-tapered before its one-sided spectrum is calculated. Let $P_{q,k}$ and $A_{q,k}$ denote the one-sided power and amplitude at positive-frequency bin k, where $k = 1, \dots, \lfloor N/2 \rfloor$.

Table S2. Equations for frequency-domain features

Feature name pattern	Equation
`{q}_dominant_frequency`	f_{k^*}, where $k^* = \arg\max_{1 \le k \le \lfloor N/2 \rfloor} P_{q,k}$
`{q}_dominant_magnitude`	A_{q,k^*}, the one-sided amplitude at the dominant non-zero frequency
`{q}_total_power`	$P_q = \sum_{k=1}^{\lfloor N/2 \rfloor} P_{q,k}$
`{q}_median_frequency`	The smallest f_k for which $\sum_{j=1}^{k} P_{q,j} \ge P_q/2$

Correlations

Pearson correlations are computed between each pair of raw axes within the window.

Table S3. Equations for correlation features

Feature name	Equation
`corr_xy`	$\frac{\sum_i (a_{x,i}-\bar{a}_x)(a_{y,i}-\bar{a}_y)}{\sqrt{\sum_i (a_{x,i}-\bar{a}_x)^2 \sum_i (a_{y,i}-\bar{a}_y)^2}}$
`corr_xz`	$\frac{\sum_i (a_{x,i}-\bar{a}_x)(a_{z,i}-\bar{a}_z)}{\sqrt{\sum_i (a_{x,i}-\bar{a}_x)^2 \sum_i (a_{z,i}-\bar{a}_z)^2}}$
`corr_yz`	$\frac{\sum_i (a_{y,i}-\bar{a}_y)(a_{z,i}-\bar{a}_z)}{\sqrt{\sum_i (a_{y,i}-\bar{a}_y)^2 \sum_i (a_{z,i}-\bar{a}_z)^2}}$

Orientation features

The three axes are low-pass filtered as continuous recordings with a second-order Butterworth filter with a 1-Hz cutoff, using forward-backward filtering. The filtered acceleration components, denoted $g_{q,i}$, are used as an estimate of the gravity vector. The mean angle is then calculated within each 1-s window. Angles are in radians.

Table S4. Equations for orientation features

Feature name	Equation
`tilt_x`	$\frac{1}{N} \sum_{i=1}^{N} \mathrm{atan2}(-g_{x,i}, \sqrt{g_{y,i}^2 + g_{z,i}^2})$
`tilt_y`	$\frac{1}{N} \sum_{i=1}^{N} \mathrm{atan2}(g_{y,i}, \sqrt{g_{x,i}^2 + g_{z,i}^2})$
`tilt_z`	$\frac{1}{N} \sum_{i=1}^{N} \mathrm{atan2}(g_{z,i}, \sqrt{g_{x,i}^2 + g_{y,i}^2})$

R code

`compute_accel_features()` accepts one uninterrupted recording of accelerometer data as a data frame or tibble with numeric `x`, `y`, and `z` columns. `fs` is the sampling frequency in Hz and must be a positive integer. `lowpass_hz` is the cutoff frequency in Hz for the continuous orientation filter and must be below the Nyquist frequency. The function returns one row per complete, non-overlapping 1-s window, containing a window index and the 54 features. Any trailing samples that do not fill a complete window are discarded.

```r
library(e1071)
library(signal)
library(tibble)

compute_accel_features <- function(data, fs = 100, lowpass_hz = 1) {
  required <- c("x", "y", "z")
  stopifnot(
    is.data.frame(data),
    all(required %in% names(data)),
    is.numeric(fs), length(fs) == 1L, is.finite(fs), fs >= 3,
    fs == as.integer(fs),
    all(vapply(data[required], is.numeric, logical(1))),
    is.numeric(lowpass_hz), length(lowpass_hz) == 1L,
    is.finite(lowpass_hz), lowpass_hz > 0, lowpass_hz < fs / 2
  )

  axes <- lapply(data[required], as.numeric)
  if (any(lengths(axes) != nrow(data)) ||
      any(!is.finite(unlist(axes, use.names = FALSE)))) {
    stop("The x, y, and z columns must contain finite numeric values.")
  }

  samples_per_window <- as.integer(fs)
  n_windows <- nrow(data) %/% samples_per_window
  if (n_windows < 1L) {
    stop("The input must contain at least one complete 1-s window.")
  }

  low_pass <- butter(2, lowpass_hz / (fs / 2), type = "low")
  gravity <- lapply(axes, function(values) {
    as.numeric(filtfilt(low_pass, values))
  })
  n <- samples_per_window
  hann <- 0.5 * (1 - cos(2 * pi * (0:(n - 1)) / (n - 1)))
```

```r
frequencies <- (0:floor(n / 2)) * fs / n
one_sided_factor <- rep(2, length(frequencies))
one_sided_factor[1] <- 1
if (n %% 2L == 0L) {
  one_sided_factor[length(one_sided_factor)] <- 1
}
frequency_step <- fs / n
output <- vector("list", n_windows)

for (window_index in seq_len(n_windows)) {
  rows <- ((window_index - 1L) * n + 1L):(window_index * n)
  window_axes <- lapply(axes, `[`, rows)
  window_gravity <- lapply(gravity, `[`, rows)
  features <- list(window = window_index)

  for (axis in names(window_axes)) {
    values <- window_axes[[axis]]
    mean_value <- mean(values)
    sd_value <- sd(values)
    prefix <- paste0(axis, "_")

    features[[paste0(prefix, "mean")]] <- mean_value
    features[[paste0(prefix, "sd")]] <- sd_value
    features[[paste0(prefix, "cv")]] <- if (mean_value == 0) {
      NA_real_
    } else {
      sd_value / mean_value
    }
    features[[paste0(prefix, "min")]] <- min(values)
    features[[paste0(prefix, "max")]] <- max(values)
    features[[paste0(prefix, "q25")]] <- unname(
      quantile(values, 0.25, type = 7)
    )
    features[[paste0(prefix, "median")]] <- median(values)
    features[[paste0(prefix, "q75")]] <- unname(
      quantile(values, 0.75, type = 7)
    )
    features[[paste0(prefix, "skewness")]] <- skewness(values, type = 2)
    features[[paste0(prefix, "kurtosis")]] <-
      kurtosis(values, type = 2) + 3
    features[[paste0(prefix, "mean_amplitude")]] <- mean(abs(values))
    features[[paste0(prefix, "sd_amplitude")]] <- sd(abs(values))
```

```r
    demeaned <- values - mean_value
    spectrum <- fft(demeaned * hann)[seq_along(frequencies)]
    raw_power <- Mod(spectrum)^2
    psd <- one_sided_factor * raw_power / (fs * sum(hann^2))
    amplitude <- one_sided_factor * Mod(spectrum) / sum(hann)
    positive_bins <- seq.int(2L, length(frequencies))
    total_power <- sum(psd[positive_bins]) * frequency_step

    if (total_power > 0) {
      peak_bin <- positive_bins[which.max(psd[positive_bins])]
      median_index <- which(
        cumsum(psd[positive_bins]) * frequency_step >= total_power / 2
      )[1]
      median_bin <- positive_bins[median_index]
      features[[paste0(prefix, "dominant_frequency")]] <-
        frequencies[peak_bin]
      features[[paste0(prefix, "dominant_magnitude")]] <-
        amplitude[peak_bin]
      features[[paste0(prefix, "median_frequency")]] <-
        frequencies[median_bin]
    } else {
      features[[paste0(prefix, "dominant_frequency")]] <- NA_real_
      features[[paste0(prefix, "dominant_magnitude")]] <- NA_real_
      features[[paste0(prefix, "median_frequency")]] <- NA_real_
    }
    features[[paste0(prefix, "total_power")]] <- total_power
  }

  features$corr_xy <- cor(window_axes$x, window_axes$y)
  features$corr_xz <- cor(window_axes$x, window_axes$z)
  features$corr_yz <- cor(window_axes$y, window_axes$z)

  gx <- window_gravity$x
  gy <- window_gravity$y
  gz <- window_gravity$z
  features$tilt_x <- mean(atan2(-gx, sqrt(gy^2 + gz^2)))
  features$tilt_y <- mean(atan2(gy, sqrt(gx^2 + gz^2)))
  features$tilt_z <- mean(atan2(gz, sqrt(gx^2 + gy^2)))

  output[[window_index]] <- features
}
```

```
  as_tibble(do.call(rbind, lapply(output, as.data.frame)))
}
```
